# Supplementary material for: Optimizing rhamnolipid biosynthesis: evaluating predictive methods using Pseudomonas aeruginosa mutants
Source: Appl Environ Microbiol. 2026 Jun 3;92(7):e00094-26. doi: 10.1128/aem.00094-26 (PMC13390351; doi:10.1128/aem.00094-26)
Supplement: Supplemental material — Figures S1 and S2; Table S1. [file aem.00094-26-s0001.pdf]

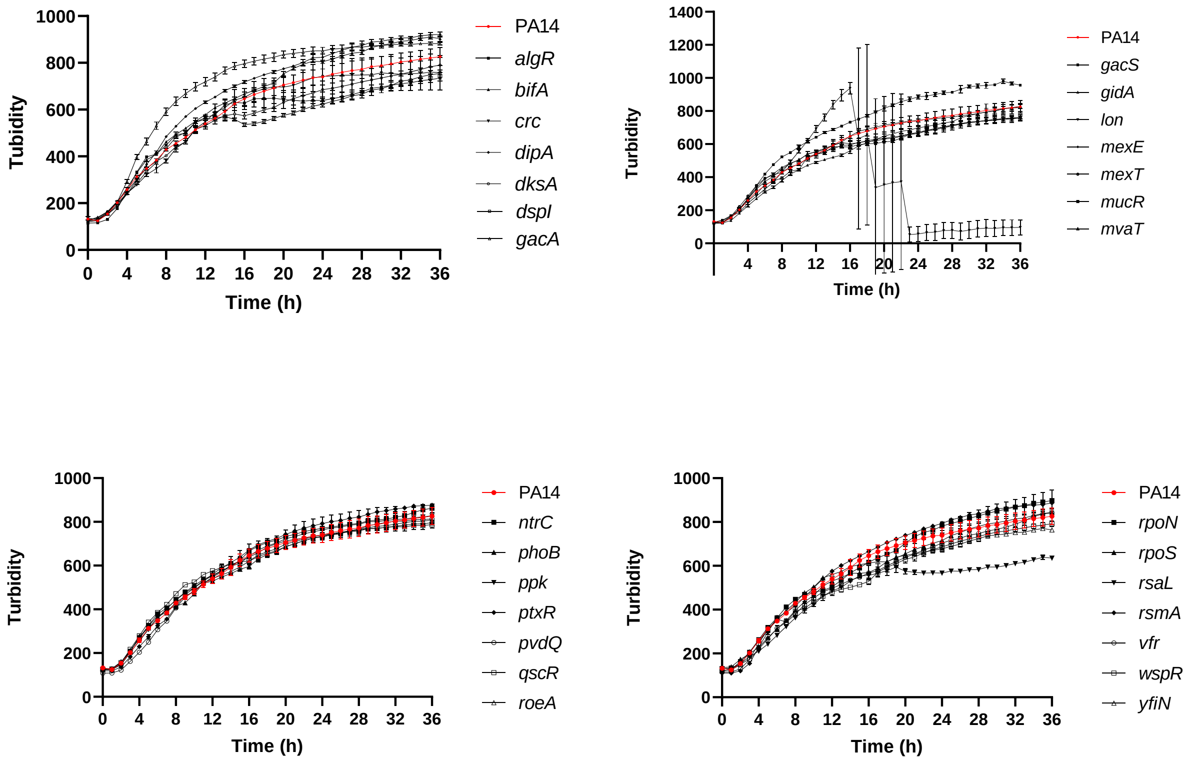

Figure S1 – Growth curves of *Pseudomonas aeruginosa* PA14 and various mutants in a Bioscreen apparatus.

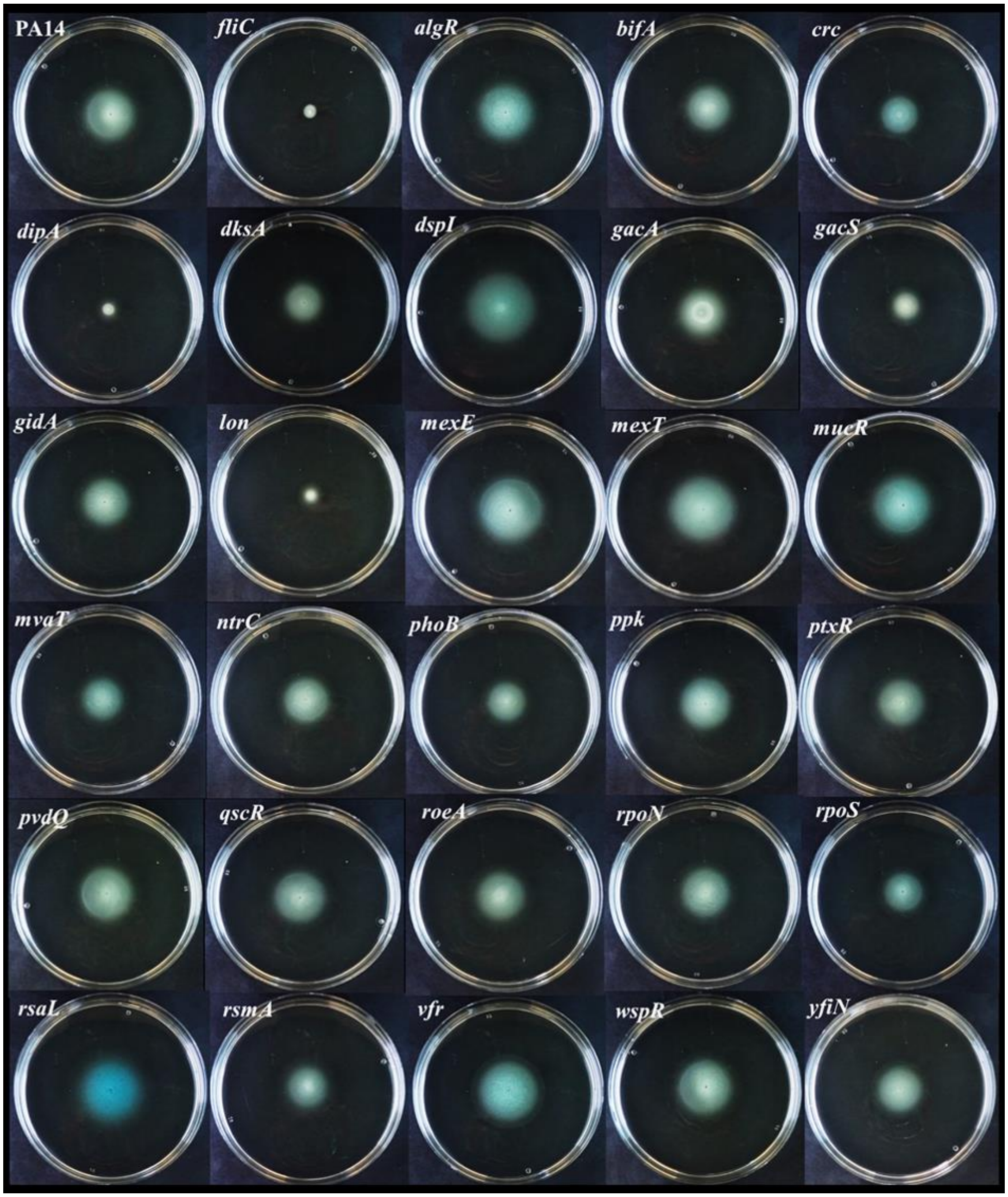

Figure S2 – Pictures of swimming motility plates of *P. aeruginosa* PA14 and various mutants.

Table S1 - Summary of swarming, swimming, Siegmund-Wagner blue plate assays, RL production (LC-MS), and biomass data.

Gene	SW blue-plate	Swarming motility	Swimming zone	Liquid cultures		
				Rhamnolipids by LC-MS	Rhamnolipids/ biomass	Biomass
algR	= SC	=	=	=	=	=
bifA	=	-	=	=	=	+***
crc	-**** SC	NF	-***	-*	-***	=
dipA	=	-	-****	=	=	=
dksA	-**** NF	-	=	-*	-***	=
dspI	-****	-	+****	-*	-**	+***
gacA	-**** SC	-	=	=	=	+**
gacS	-*	-	-****	=	=	=
gidA	=	=	=	=	=	=
lon	-** SC	NF	-****	NT	NT	NT
mexE	=	=	+**	=	=	=
mexT	=	=	+****	=	=	=
mucR	=	=	+****	=	=	=
mvaT	= SC	=	-****	=	=	=
ntrB	-**** SC/NF	=	=	=	=	=
ntrC	-**** SC/NF	=	-****	=	=	=
phoB	=	=	-****	=	=	=
ppk	=	=	=	=	=	=
ptxR	=	=	-*	=	=	=
pvdQ	=	-	=	+*	=	=
qscR	=	=	=	=	=	=
roeA	=	=	=	=	=	=
rpoN	=	-	=	+*	=	+****
rpoS	-****	-	-***	=	=	=
rsaL	-**** SC/NF	-	=	NT	NT	NT
rsmA	-**** SC	-	-***	=	=	=
vfr	-**** SC	=	=	=	=	=
wspR	=	=	=	=	=	+***
yfiN	=	=	-*	=	=	=

Abbreviations: NF = not formed; SC = small colony; NT = not tested. Symbols: "+" = higher than WT; "=" = equal to WT; "–" = lower than WT. Data were analyzed by one-way ANOVA followed by Dunnett's post hoc test vs. control (****$p < 0.0001$, ***$p < 0.001$, **$p < 0.01$, *$p < 0.05$).
